# In it for the long run: perspectives on exploiting long-read sequencing in livestock for population scale studies of structural variants

**DOI:** 10.1186/s12711-023-00783-5

**Published:** 2023-01-31

**Authors:** Tuan V. Nguyen, Christy J. Vander Jagt, Jianghui Wang, Hans D. Daetwyler, Ruidong Xiang, Michael E. Goddard, Loan T. Nguyen, Elizabeth M. Ross, Ben J. Hayes, Amanda J. Chamberlain, Iona M. MacLeod

**Affiliations:** 1 Agriculture Victoria, AgriBio, Centre for AgriBioscience, Bundoora, VIC 3083 Australia; 2 School of Applied Systems Biology, La Trobe University, Bundoora, VIC 3083 Australia; 3 Faculty of Veterinary & Agricultural Science, The University of Melbourne, Parkville, VIC 3052 Australia; 4 Queensland Alliance for Agriculture and Food Innovation, University of Queensland, St Lucia, QLD 4072 Australia

## Abstract

Studies have demonstrated that structural variants (SV) play a substantial role in the evolution of species and have an impact on Mendelian traits in the genome. However, unlike small variants (< 50 bp), it has been challenging to accurately identify and genotype SV at the population scale using short-read sequencing. Long-read sequencing technologies are becoming competitively priced and can address several of the disadvantages of short-read sequencing for the discovery and genotyping of SV. In livestock species, analysis of SV at the population scale still faces challenges due to the lack of resources, high costs, technological barriers, and computational limitations. In this review, we summarize recent progress in the characterization of SV in the major livestock species, the obstacles that still need to be overcome, as well as the future directions in this growing field. It seems timely that research communities pool resources to build global population-scale long-read sequencing consortiums for the major livestock species for which the application of genomic tools has become cost-effective.

## Background

Many studies in livestock have exploited variation at the sequence level to understand population-scale diversity and for the genetic improvement of livestock. However, most of these studies were restricted to single nucleotide polymorphisms (SNPs, or single nucleotide variants—SNV), and small INsertions/DELetions—INDEL (< 50 bp) that can be detected confidently with short-read sequencing. Genomic variations that involve a longer segment of DNA, i.e. more than 50 bp, are referred to as structural variants (SV) [1] and have not yet been extensively studied in livestock, and particularly not at the genome-wide and population scales. In general, there are two types of SV, either balanced (such as inversions or translocations), or unbalanced (such as insertions, deletions, or copy number variations [CNV]). Previous studies on the human genome have estimated that structural variations represent a proportion of the total genome that could be equal to or exceed that of SNPs and small INDEL [2, 3]. In the bovine species, ~ 3.1% (94.4 Mb) of the genome was estimated to consist of segmental duplications (≥ 1 kb long and with ≥ 90% sequence identity) [4] and these regions typically harbour many CNV [5]. A later analysis has shown that up to 10% of the bovine genome may contain deletions and tandem duplications [6]. A study that was published in 2021 [7] assembled a pangenome from only six bovine genomes and revealed 70.3 Mb of non-reference SV when compared to the standard bovine reference genome (assembled from a single animal).

Structural variation in the genome can have a direct or indirect influence on both complex and Mendelian phenotypic variation through multiple mechanisms, such as the alteration of the DNA sequence in regulatory or functional gene regions [8–10]. In spite of their importance, SV remain much more poorly considered than their smaller mutational counterparts, mainly due to the difficulty in characterising such regions using the short-read sequencing technology, and have been described as biological dark matter [11]. Since the advent of high-throughput genomics in the early 2000s, multiple attempts using mainly the short-read technology have been made to characterize SV that potentially may be causal variants for defects, diseases, or other traits in the major livestock species that have good quality reference genomes (Table 1). Interestingly, some of these CNV detected by analysing short reads have begun to be included on SNP arrays, however the use of SNP arrays to characterize/discover SV is out of the scope of this study. While the short-read technology (also known as 2^nd^ generation sequencing) has provided a cost-effective and accurate means of detecting small variants (< 50 bp), its limitation of the length of the short reads has made it technically challenging to accurately detect large SV as well as SV located in tandem repeat rich regions. The so-called 3^rd^ generation sequencing technologies (or long-read sequencing) are much more appropriate to directly address the identification of SV [12]. Recent studies have highlighted that a substantial proportion of previously hidden structural variation can be discovered with long-read sequencing [7, 13] through technological advancements that enable huge fold increases in read lengths compared to 2^nd^ generation sequencing (typically longer than 10 kb). Although in the past, the per base accuracy of 3^rd^ generation long-read sequencers was not comparable with that of Illumina short-read sequencing [11], the ongoing development of cutting-edge chemistry [14] as well as software development [15] are rapidly addressing this issue. In addition, improvements in dry/wet lab methods have been published over recent years to promote the use of long reads that improve the continuity, accuracy, and range of variant calling/processing as well as* de novo* assemblies [16].

**Table 1:** Structural variant discoveries using a “focused approach” in livestock, using either short-read (SR) and/or long-read (LR) sequencing

Phenotype/disease name	Species	Breed	Sequencing platform	Summarized of key findings	Ref.
Recessive lethal and increased milk production	Cattle	Nordic Red	SR	A 660-kb deletion on chromosome 12 encompassing four genes is believed to be the putative recessive causative variant, and results in embryonic death; this outcome is thought to result from the loss of the* ribonuclease H2, subunit B* (*RNASEH2B*) gene	[51]
Polled	Cattle	Friesian	SR	An 80-kb duplication on chromosome 1 was found to cause a dominant poll phenotype in Friesian cattle; it was confirmed in 6000 animals that were genetically tested for the polled phenotype	[87]
Cholesterol deficiency	Cattle	Holstein	SR	A 1.3-kb insertion on chromosome 11 of a transposable long terminal repeat element (ERV2-1) in the *APOB* gene was reported to cause early death in Holstein cattle	[88]
Muffs and beard (Mb) phenotype	Chicken	Multiple breeds	SR	A complex SV (three duplications, one in tandem and two that are translocated to that of the tandem repeat on chromosome 27) was found to have an incomplete dominant effect on the *Mb* phenotype in chicken; this SV leads to continuous high ectopic expression of the *HOXB8* gene in the facial skin	[89]
Holstein lethal haplotype 5 (HH5)	Cattle	Holstein	SR	A 138 k-bp deletion on chromosome 9, covering the *dimethyl-adenosine transferase 1* (*TFB1M*) gene was reported to have a recessive effect causing embryonic death	[90]
Finching or line-backed spotting	Cattle	Pinzgauer	SR and LR	A complex structural variant consisting of a 9.4-kb deletion and an inversely inserted 1.5-kb duplication fused to a 310-kb duplicated segment from chromosome 4 was confirmed to be causative and dominant for the phenotype	[91]
Tetradysmelia	Cattle	Holstein Friesian	SR	A 50-kb deletion on chromosome 14 identified in several members of a Holstein–Friesian family, which most likely disrupts the bovine *R-spondin 2* (*RSPO2*) gene, causing the autosomal recessive condition (tetradysmelia)	[92]
Colour-sidedness	Cattle	Gloucester	SR	A complex structural variant, caused by two related translocations (chromosomes 6 and 29) downstream of the *KIT* gene; all three alleles potentially disrupt several putative regulatory elements downstream of *KIT*, that result in the dominant colour-sidedness phenotype	[93]
Mastitis resistance	Cattle	Holstein Friesian	SR	A 12-kb multi-allelic CNV on chromosome 6 covering the *GC* gene enhancer is associated with mastitis resistance in dairy cattle and *GC* gene expression	[50]
Bulldog calf syndrome (BDS)	Cattle	Holstein	SR	A 3513-bp deletion on chromosome 5, spanning 10 coding exons of the *collagen type II alpha 1 chain* (*COL2A1*) gene was reported as the causative variant for BDS, a dominant inheritance lethal syndrome	[94]
Polled	Cattle	Brahman	LR	Long read sequencing study revealed a 212-bp insertion in place of a 10-bp deletion on chromosome 1 in Brahman poll animals; this structural variant was found to be absent in horned animals	[95]
Polled intersex syndrome (PIS)	Goat	Multiple breed	SR and LR	A complex structural casual variant consisting of a 10,159-bp deletion and an inversely inserted 480-kb-sized duplication on chromosome 1; these regions appeared to span across two functional genes resulting in a dominant female intersex phenotype	[96]
X-Linked hypohidrotic ectodermal dysplasia	Cattle	Red Angus-Simmental	SR	A 53-kb deletion of the X chromosome that includes a section of the *ectodysplasin* (*EDA*) gene as well as the entire *acyl-CoA wax alcohol acyltransferase 2* (*AWAT2*) gene; it was reported in a family of cattle diagnosed with X-linked hypohidrotic ectodermal dysplasia	[97]
Coat color darkening	Cattle	Nellore and Brahman	SR and LR	A complex structural rearrangement consisting of a 1155-bp deletion combined with an insertion of more than 150 bp including a SINE element seemed to be the most plausible causal candidate due to its size and location near the *agouti signalling protein* gene (*ASIP*) on chromosome 13	[98]
Plumage colour	Chicken	Rhode Island Red crossed to White Leghorn	SR	A 7.6-kb deletion in the non-coding region upstream of the *SOX10* gene responsible for light yellow/dark brown plumage	[99]

*LR* long read, *SR* short read

To date, the main focus of the SV investigations in livestock has been the characterization and application of CNV [4, 5, 17–22]. In general, there has been strong interest in the discovery of SV in livestock (see Tables 1 and 2). As a direct result of the technological limitations of short-read sequencing as well as the cost of building large reference populations with long-read sequences, currently two key elements for the detection of SV in livestock are missing:

1. Genome-wide population scale SV discovery and imputation.
2. Studies to determine associations between genome-wide SV and quantitative traits (a previous attempt using short-read information highlighted the difficulties of this approach [23]).

**Table 2:** Structural variants detected in livestock based on two discovery approaches (RS: resequencing or PG: pangenome approach) with either short-read (SR) or long-read (LR) sequencing technology

Application	Species	Breed	Technique | sequencing technology	Brief summary of key findings	Ref.
Genome-wide study of SV in dairy breeds	Cattle	Holstein, Montbéliarde, Normande	RS | SR	This study discovered 6426 putative structural variants that segregated in whole-genome sequence data in a total of 62 bulls representing three major French dairy breeds	[6]
Detection of SV by large-scale sequencing revealed evolutionary evidence of breed divergence between Chinese and European pigs	Pig	Multiple breeds	RS | SR	56,930 putative SV were discovered using genomes of 13 pigs from diverse breeds that originated from China and Europe	[100]
CNV in the sheep genome	Sheep	Multiple breeds	RS | SR	A study of CNV in sheep, using multiple methods to identify and characterise copy number changes, resulted in 3488 autosomal CNV regions with an average length of 19 kb	[18]
Detection of a reciprocal translocation in a pig line	Pig	Large-White line	RS | SR	A resequencing approach identified a translocation variant occurring within the coding region of the gene and results in a loss of expression of a disintegrin and metalloproteinase with thrombospondin motifs (ADAMTSL4), but no observable phenotype was detected with this translocation	[101]
Genome-wide mapping of large deletions and their population-genetic properties in dairy cattle	Cattle	Holstein, Jersey, Nordic Red cattle	RS | SR	This study reported 8480 large deletions (199 bp–773 kb) from 175 cattle	[102]
Detection and assessment of CNV using PacBio long read and Illumina sequencing in New Zealand dairy cattle	Cattle	Holstein–Friesian	RS | SR and LR	One LR sequenced and 556 SR sequenced New Zealand cattle revealed little overlap between the two platforms; PacBio sequencing resulted in 38,709 putative SV, of which 19,797 were CNV, while Illumina sequencing resulted in 43,708 putative CNV	[103]
Identification of CNV in domestic chicken with short read sequencing	Chicken	Multiple breeds	RS | SR	Mining whole-genome sequences of 65 chickens from multiple breeds detected 663 domestication-specific CNVR across the autosomes	[104]
CNV in Holstein–Friesian cow genomes based on whole-genome sequence data	Cattle	Holstein–Friesian	RS | SR	Thirty-two sequenced Holstein–Friesian cows were analyzed to evaluate the landscape of CNV; length of deletions ranged from 5234 to 9145 bp and the average length of duplications ranged from 7254 to 8843 bp, but most of the CNV were unique to a single cow although several were validated in previous studies	[105]
Database of CNV discovered in taurine cattle	Cattle	Multiple breeds	RS | RS	More than 500 bulls from 17 breeds were used to reveal 26,223 CNVR covering 107.75 unique Mb of the bovine genome; this study also confirmed the genotypes of a CNVR at the *KIT* locus associated with the piebald coat colour phenotype in Hereford and Simmental cattle	[106]
Comparative analyses of CNV between *Bos taurus* and *Bos indicus*	Cattle	Multiple breeds	RS | SR	This study identified 13,234 non-redundant CNV regions in 73 animals from 10 cattle breeds (4 *Bos taurus* and 6 *Bos indicus*)	[107]
CNV in Chinese indigenous cattle	Cattle	Multiple breeds	RS | SR	Resequencing approach using genomes from 75 cattle individuals (including six Chinese indigenous cattle breeds and two non-native specialized beef cattle breeds) discovered 11,486 CNVR; many of these are related to traits of interest including meat production or quality, coat color, adaptation	[108]
Identification of CNV in Holstein dairy cattle from whole-genome sequence and genotype array data	Cattle	Holstein	RS | SR and genotype	SR sequence and genotype arrays of 96 Holstein animals revealed high confidence CNV regions that overlapped using both methods	[109]
Haplotype-resolved genomes in Angus and Brahman cattle	Cattle	Angus × Brahman cross	PG | LR	Chromosome-level genome assemblies of Angus and Brahman cattle revealed structural and copy number variants that may be subspecies specific; the study estimated that the total lengths in bp affected by SV in Angus and Brahman were 10.9 and 21.8 Mb, respectively	[66]
SV in cattle	Cattle	Multiple breeds	RS | SR	This study applied sequencing approaches with 72 whole-genome sequences representing taurine and indicine cattle; 16,738 SV were identified, of which 1575 were novel	[110]
Novel functional sequences uncovered through a bovine multi-assembly graph	Cattle	Multiple breeds	PG | LR	A pangenome approach to study SV using six genome assemblies from taurine and indicine cattle as well as yak identified 68,328 SV present in the multi-assembly graph	[7]
Analysis of SV in Meishan pigs	Pig	Multiple breeds	RS | SR	Structural variants in Meishan pigs together with genomic data from 55 pig breeds from Africa, Europe, Asia, South-east Asia, and Central America, and included wild boars, this study curated 33,698 SV in 305 individuals	[111]
Detection and validation of SV in bovine whole-genome sequence data	Cattle	Holstein and Jersey	RS | SR	Detection and validation of SV in bovine whole-genome sequence data from 252 Holstein and 64 Jersey bulls with 17,518 SV in Holstein and 4285 SV in Jersey	[23]
CNV in Chinese indigenous fine-wool sheep populations using whole-genome resequencing	Sheep	Multiple breeds	RS | SR	Genomic CNV map for Chinese indigenous fine-wool sheep from 32 fine-wool sheep from three breeds were analyzed using resequencing approach; the analysis curated 1,747,604 CNV and 7228 CNV regions, which were estimated to represent 2.17% of the sheep reference genome	[112]
SV relate to domestication in small ruminants (goats and sheep)	Goat and sheep	Multiple breeds	RS | SR	Study aimed at investigating the role of SV during the domestication and subsequent genetic improvement of goats and sheep that revealed 45,796 SV in the sheep and 15,047 SV in goat genomes, respectively	[113]
Chicken pangenome revealed a SV catalogue and a causal variant for body size	Chicken	Multiple breeds	RS | SR	A pangenome approach using short read sequencing of 664 individuals discovered 66.5 Mb of sequences that are absent from the genome assembly; this study identified deletions on Chr 27 in the promoter region of *IGF2BP1* that affects body size in chickens	[114]
Bovine pangenome reveals trait-associated structural variation from diverse assembly inputs	Cattle	Multiple breeds	PG | LR	A pangenome approach revealed 900 SV overlapping with coding sequences; these included variants affecting the *QRICH2*, *PRDM9*, *HSPA1A*, *TAS2R46* and *GC* genes that can affect phenotypes	[115]
Initial study of analysing SV detections in cattle	Cattle	Unknown breed	RS | LR and SR	One individual sequenced using 10× Genomics linked read, PacBio as well as ONT; the study identified 21,164 SV, which amount to 186 Mb covering 7.07% of the whole genome	[116]
Sheep graph-based pangenome reveals the spectrum of SV and their effects on tail phenotypes	Sheep	Multiple breeds	PG | LR	This study characterized 26 haplotype-resolved genome assemblies from 1342 genetically diverse sheep and performed a graph-based approach to genotype biallelic SV in 684 individuals from 45 domestic sheep breeds and two wild species; it resulted in 142,422 biallelic insertions and deletions, 7028 divergent alleles and 13,419 multiallelic variations	[117]
Novel SV and missing sequences providing new insights into bovine diversity and evolutionary history	Cattle	Multiple breeds	PG | LR	A pangenome approach using 898 cattle from 57 bovine breeds identified 83 Mb of sequence not found in the cattle reference genome (~ 3% different from the reference genome). A catalogue of the SV revealed 3.3 × 10^6^ deletions, 0.12 × 10^6^ inversions, and 0.18 × 10^6^ duplications	[118]
Short read sequencing to characterise balanced reciprocal translocations in pigs	Pig	Unknown breed	RS | SR	Deployment of a structural variant calling software and paired end short-read sequencing with a depth of at least 20-fold coverage that detected and characterized balanced reciprocal translocations in 7 carriers but did not detect any such translocation in 15 non-carriers; the results suggest that paired end short read sequence data can be used to detect and characterize balanced reciprocal translocations, but may be limited in the detection of translocations in repetitive regions	[119]
Detection of SV	Chicken	Multiple breeds	RS | LR and SR	Sequencing of ten chickens from various breeds using the PacBio technology detected 49,501 high-confidence SV	[120]

*SV* structural variants, *CNV* copy number variants, *SR* short-read, *LR* long- read, *PG* pangenome, *RS* resequencing

Curation of large reference populations with long-read sequences is essential to address both elements (1) and (2). Cataloguing SV and their frequency spectrum in each population using long-read technology is a critical first step towards: understanding the extent of this variation, imputing SV into larger genotyped populations, and undertaking further downstream research (e.g., interpretation of breed diversity, association with a range of phenotypes such as disease susceptibility, environmental adaptation, etc.). It is important to mention that due to differences in the structure of breeding programs from one species to another, the strategies to deploy genetic improvement can be specific to each type of livestock. However, the overarching framework is still most likely to be “Discover + Impute $\Rightarrow$ Impact”.

Previously, in 2014, the landscape of SV in livestock as well as the challenges in this field of study were reviewed [22]. However, with the rapid advances in long-read sequencing since then, as well as the recent progress in the field of bioinformatics, we consider that it is timely to provide here updated perspectives on:

1. The progress of the methods and strategies for genome-wide SV discovery in livestock species where genomic tools are routinely available (cattle, sheep, goats, pigs, and chicken).
2. The challenges and prospects for population-scale discovery and application of genome-wide SV for livestock breeding.

In the last decade, the development of technologies for 2^nd^ generation sequencing has been dominated by Illumina. Their sequencing technology is highly cost-effective with high base-calling accuracy and well supported downstream analysis tools and pipelines [24]. Another advantage of 2^nd^ generation sequencers is that the library preparation itself does not require high-quality DNA. Libraries can be prepared with short DNA fragments, even ancient DNA that is highly degraded. However, the key technical feature of 2^nd^ generation short-read sequencers is that they only provide reads with a limited read length: generally, less than 300 bp. These short reads have minimal potential to identify (i) large SV, because the short reads that are derived from them are difficult to accurately map to a reference genome, and (ii) SV within repetitive sequences such as large segmental duplications, which may not be resolved with short-read mapping algorithms. It should be noted that even for the discovery of small variants in chromosomal regions with large segmental duplications, short reads result in much lower accuracy than long reads because of difficulties of their alignment in these regions [25].

In an effort to improve the detection of SV using short reads, several studies have relied on a technology that creates “virtual long reads” to further increase read length with techniques such as: mate-pair reads [26, 27], linked-read technologies from 10X Genomics [28], MGI single-tube long fragment read (stLFR) [29], or Illumina’s recently announced long-read sequencing assay, i.e. complete long read (CLR) at the time when this manuscript was written, November 2022. These approaches can theoretically extend read length while maintaining the low base call error rate and cost efficiencies. However, many of these technologies are still under development and can be considered as “advanced short-read sequencing” instead of “long-read native DNA sequencing”. In addition, in the last few years, multiple studies have performed a combination of short-read sequencing with several other add-on technologies, for example, with long-read sequencing as well as optical mapping (Bionano Genomics) or Hi-C sequencing techniques to greatly enhance the ability to find and validate SV at the genome level [30–33].

Evolving from short-read sequencers, the development of 3^rd^ generation sequencers began in the early 2000s with key competitors including Pacific Biosciences (PacBio) with single-molecule real-time (SMRT) sequencing and Oxford Nanopore Technologies (ONT) developing nanopore sequencing. Although they are in the same wave (3^rd^generation sequencers), PacBio and ONT differ widely in their principle of action. Nanopore sequencers measure the ionic current fluctuations when single-stranded nucleic acids (DNA/RNA) pass through biological pores (so-called ‘nanopores’) [34]. The read lengths with ONT vary with the input fragment lengths, therefore the term “N50” is often used to describe the read length where 50% of the data is contained within reads with lengths greater than the N50 value. Typically, ONT sequencing achieves N50 of more than tens of thousands of kb and it is possible to reach maximum lengths of several Mb (the longest recorded is 4 Mb [35]). In contrast to ONT, PacBio sequencers use fluorescence polymerase tethered to the bottom of a well to predict nucleic acid sequences [36]. Their high fidelity (HiFi) read lengths are typically around thousands of kb (10–25 kb, [37]) with very high accuracy. At the time this article was written, through various optimizations in the workflow, PacBio HiFi reads have achieved a per base quality score accuracy that nearly equals that of Illumina short reads [38]. In the past, several studies in the field of genomics have reviewed long-read sequencing technologies, its opportunities and limitations [11, 12], as well as performed benchmarking across multiple technologies [39]. Undoubtedly, now and in the near future, these technologies will continue to be developed to further increase yield, base call accuracy, maximum read length while reducing overall sequencing cost [40].

With long-read sequencing, there are currently two major approaches to detect genome-wide SV in multiple individuals, the first uses the “assembly” method to generate a “pangenome”, and the second uses a so-called “resequencing” approach, with the potential to combine both:

1. The assembly/pangenome method generally applies a de novo assembly approach to the sequence of each individual (i.e., no prior reference genome is used for alignment) and aims at generating a haplotype-resolved pangenome. The de novo approach enables SV to be identified using a compare and contrast method between multiple assemblies and removes the inherent bias when using a reference genome from a single individual of a particular breed. The aim of a pangenome approach is ultimately to provide a new reference genome that is not limited to a single individual but encompasses a much broader range of the structural variation that exists across a species. Thus, the approach is generally undertaken with a limited number of individuals each from diverse populations (e.g., breeds). In addition to providing a pangenome reference, this expands the knowledge on the extent of unique structural variation across diverse individuals and enables a more complete annotation of genes and transcripts using long-read sequencing [41]. For the bovine species, the Bovine Pan Genome Consortium (PBC) has begun important work in creating a pangenome using individual animals from very genetically-diverse breeds, sub-species and species while also cataloguing the extent of SV discovered (https://bovinepangenome.github.io/).
2. On the other hand, the resequencing approach uses sequencing reads from an individual and aligns these against a specified reference genome that is generally derived from a single individual. Following alignment, the different sites between the new and reference sequence can then be assessed at an individual level as well as at a population level. In general, the key aim of the re-sequencing approach is to detect variation in a significant number of individuals (potentially all from the same population) with a view to then linking the genomic variation to specific phenotypes and evolutionary processes.
3. Ideally, in the foreseeable future, the reference genome for the resequencing approach can be assembled from multiple animals and will be either population (breed) specific or a pangenome. Although software tools have been developed to align reads and call variants using a pangenome reference (e.g., Pangenie [42], Vg [43], and Giraffe [44]), improved efficiency and compatibility are required to become feasible at the population scale with long-read sequences [41].

Due to the exacting sequence quality requirements for de novo haplotype-resolved assembly, the accuracy of SV discovery from the pangenome will outperform the re-sequencing approach [45]. However, the assembly approach will be considerably more costly on a per individual sequence level compared to the re-sequencing approach because: (i) de novo assembly requires high-sequencing depth (50–60× with older long-read technologies, and trending towards 20–30× with latest releases), while the re-sequencing approach may compromise with lesser coverage (Nguyen et al., unpublished); (ii) ideally the parents of the individuals used for the pangenome assembly are also sequenced (often with short-read technology) to enable the required resolution of haplotypes; and (iii) the additional sequencing results in significantly higher computing costs compared to the re-sequencing approach.

The above descriptions demonstrate that these two approaches for the discovery of SV are complementary, such that in the future, as pangenome references and improved bioinformatics tools become available for resequencing studies, this will greatly expand the repertoire of SV detected at the population scale. Thus, in addition to pangenome development, livestock improvement applications will require the discovery of SV across many individuals within specific populations, to catalogue the level of SV diversity within breeds and to build reference populations for downstream analyses. The resequencing approach allows for a more cost-effective sequencing of a larger number of individuals, which then enables studies such as association of SV with specific phenotypes, either directly or through imputation of SV into even larger populations that are already genotyped with dense SNP panels, short-read sequencing or low pass long-read sequencing. The first successful example of a population-scale SV study (discovery, imputation and association) was in a human Icelandic population where SV were found to be associated with complex traits [10].

## Recent examples of SV studies in livestock

To date, sequence-based studies of SV in livestock (short and long reads) have implemented two main approaches: one is a “focused approach”, where a priori, a phenotype is tracked and then associated with SV in a genomic region of interest (summarized in Table 1), and the other is a naive “discovery approach” (summarized in Table 2). In the latter, multiple SV can be identified from genome-wide scanning using either (a) a resequencing or (b) a pangenome method. In Tables 1 and 2, we summarize recent studies using these two methodologies in several key livestock species where genomics tools are well developed (cattle, sheep, goat, pig, and chicken), because there have been many developments since the last major review on the SV landscape in livestock [22].

## Perspectives on the importance of SV for livestock improvement

Due to their large size, SV are known to influence gene function, as they might cause partial/complete gene knockout or even may alter gene expression of neighbouring genes: this phenomenon is confirmed in humans [46], plants [47] and animals [48]. Currently, the SV that have been identified in livestock as putatively causal, are biased towards those that have a large monogenic influence on a phenotypic trait, but some have also been identified as affecting quantitative traits (see examples in Tables 1 and 2). In the past few years since the advent of cheaper sequencing, a range of monogenic traits involving SV have been dissected using the focused approach in multiple livestock species (Table 1). However, in general, causal variants that underpin a physical defect/feature or inherited Mendelian disease including recessive lethal mutations in livestock are often not confirmed at the molecular level. There are numerous reasons for this, including the high investment cost (R&D, sequencing, and turnover time), difficulties capturing genetic material (farm to laboratory distance, rarity of cases, short lifespan of the embryo/animal, and producer’s concerns over reputation). For quantitative traits in livestock, it has been even more difficult to unequivocally identify any type of causal variant due to the large numbers of individuals required to detect the generally smaller effects and also due to strong linkage disequilibrium between variants extending over long distances (often several hundred kb) [49]. To date in livestock, there are few published examples of putative causal SV affecting complex traits, although there are two interesting examples in cattle (a CNV and a large deletion) that appear at a moderate frequency and have antagonistic pleiotropic effects on important traits [50, 51].

Clearly, to have adequate power to detect associations with quantitative traits it is necessary to be able to generate large numbers of individuals with real/imputed SV genotypes and phenotypes. This approach has already been applied with some success in plants [52], yeast [53] and humans [10, 54]. The evidence from such studies indicated that there may be high value in developing the catalogue of SV in reference populations of livestock, imputing, and testing the effects of these variants in large populations of animals with recorded traits, and applying these findings to breed improved livestock. The main challenges that need to be addressed fall into three main areas: (i) developing large long-read sequenced reference populations to enable effective and accurate SV discovery and imputation; (ii) evaluate molecular mechanisms that underpin SV effects on phenotypic traits; and (iii) apply knowledge of SV location and genotype to improve genomic tools for animal breeding.

## Developing large long-read sequenced reference populations to enable effective and accurate SV discovery and imputation

### Building long-read reference populations for SV discovery, phasing and imputation

We propose that it is timely to begin large collaborative long-read sequencing projects for livestock species using the cost-effective re-sequencing approach, similar to the existing short-read collaborations (e.g., 1000 Bull Genomes Project and SheepGenomesDB). Ideally, similar to the 1000 Bull Genomes Project, the reference populations would include: (i) at least hundreds of individuals for each of the most numerous breeds because the rarer are the variants the more individuals are required for discovery and accurate imputation; (ii) small numbers of rarer breeds and outspecies; (iii) popular common ancestors of the current population where possible; and (iv) at least 10 or more trios (offspring and parents) for targeted studies including bioinformatic quality control.

Within each species, we consider that there should be close collaboration between pangenome, long-read and short-read consortiums because this would enable the most effective use of the different levels of genomic information available, for example:

1. Deeply sequenced pangenome animals can be used: in the short-term to augment the size of the sequenced population, and in the medium- to long-term to be deployed as a breed-specific or pangenome reference for alignment of re-sequencing long read data.
2. Existing short read databases with many sequenced individuals would continue to be invaluable for imputation of small sequence variants (e.g., 1000 Bull Genomes Project now includes over 9000 genomes), some individuals for which short reads and DNA are still available could be added to the long-read reference to provide individuals (such as trios) for specific studies such as: testing SNPs, INDEL and SV discovery/imputation, testing new bioinformatic tools that use short reads for the discovery of some types of SV, including tools that rely on high confidence SV sets that will become available from the long-read work (e.g., Giraffe, PanGenie).
3. In the short- to medium-term, a high-confidence set of SV in specific populations could be documented through long-read SV discovery (pangenome and/or re-sequencing). This ‘truth set’ could be used for a range of purposes including its use with short-read sequence databases for improved SV detection, although this will necessarily have considerable biases such as tending to exclude SV in segmental duplication regions [10]. However, where population-scale short-read sequence databases already exist, this might enable some limited population-scale SV detection and imputation, while long-read sequence databases are being developed.

One of the main weaknesses of long reads in the past few years was the single base accuracy, and previous studies have suggested that this might lead to incorrect small variant calling [36, 55, 56]. This resulted in the development of approaches such as hybrid base-call correction for long reads using short reads (‘polishing’) to improve the single base accuracy [57, 58]. However, at the time this article was written (November 2022) and looking forward, the likely verdict is that single base errors will become a non-issue. This is because the field is rapidly progressing in many aspects (technologies and bioinformatics), such as the most recent high accuracy PacBio developments (including HiFi) as well as ONT R10.4 flow cells that claim dramatic improvement in per base accuracy, bringing new advances that could result in high-quality small variant calls equivalent to short -read technology [37, 38, 59]. This means that SNPs and small INDEL variants called in long-read re-sequencing could be added to existing short-read variant databases to augment the data available for their imputation. Furthermore, although it is critical to maintain and provide access to these short-read databases, there would be no need to go on increasing the size of the short-read sequence database in populations that have the resources to undertake long-read sequencing. Arguably, for livestock species that do not yet have a short-read sequence database, there would no longer be a need to develop a short-read database if resources could be switched to sequencing adequate numbers of individuals with long reads.

A considerable strength of long-read sequencing is the relative ease for deployment of read-based, long-range haplotyping (instead of the traditional haplotype phasing), where phase information present in the reads can be incorporated into algorithms as true data to calibrate phasing and imputation models. This has been adopted in several recent phasing and imputation algorithms, for example: WhatsHap [60], HapCUT2 [61], QUILT [62], Duet [63] or LongPhase [64]. This should enable improved imputation (which relies on accurate phasing) of SV using long-read data compared to using short-read data and this was confirmed in a human study [10]. In the past, we have demonstrated that imputation accuracy for SNPs and small INDEL is improved by combining short-read sequence from multiple breeds and crosses [65]. However, it is yet to be determined if this will still hold for the imputation of SV using long reads, and should it not be the case, it could necessitate increased numbers of individuals that are sequenced within a breed.

### De novo assemblies to build pangenomes

Assuming that the sequencing cost of long-read technologies will continue to significantly decrease in the near future, it would be useful to perform high read depth sequencing and construct pangenome scale assemblies. Recent studies in humans and bovine have identified that hundreds of Mb of the population- and individual-specific sequences are absent from the reference genome [7, 66] and it is therefore likely to be the case in other livestock species. Therefore, as discussed above, planning for de novo assemblies with long reads is desirable to create breed-specific or pangenome references, as well as to gain deeper insights into evolutionary modifications and comparative functional genomics between breeds and individuals. However, given the high costs per animal to undertake haplotype-resolved de novo assembly, if resources for long-read sequencing are limited in a given species, then it could be more cost-effective to initially focus only on building a consortium that undertakes a re-sequencing approach with the current reference genome. This will build a long-read sequence population, while waiting for improvements in cost-efficiency before developing breed-specific or pangenome references. At a later stage, it would be possible to redo the re-sequencing alignment to a breed-specific or pangenome reference to improve on the initial SV discovery.

## Validation of SV effects and evaluating their role in molecular mechanisms

### Biological validation of specific SV

Currently, wet-lab methods can be employed to validate SV post-discovery, for example, some available options include: (i) long-range PCR amplification in combination with gold standard Sanger sequencing or (ii) Bionano optical mapping can be considered a cost-effective method. In addition to this approach, long-read sequencing of parent–offspring groups can also provide a means to confirm SV inheritance patterns to validate the presence of SV [67]. Once SV from individuals have been confirmed to be accurately predicted and putatively causal, it is of great interest to undertake biological investigations to reveal the molecular mechanisms that underpin the effect of SV on important traits in livestock. Then for example, a functional approach such as knockout via gene silencing or CRISPR might be considered for downstream validation. However, it is important to note that these validation methods are often low-throughput, so there is a necessity for the further development of higher throughput validation methods for SV similar to the deployment of massively parallel reporter assays (MPRA) in SNP functional confirmation [68].

### Genome-wide validation of SV effects

Similar to SNPs, SV may have the potential to affect promoter/enhancer activity, alter gene expression, and in some case, cause malfunction/fusion of genes by combining/separating genomic regions together or separating a genomic region into sub-regions. Therefore, it would be of great interest to test the effect of SV on gene expression through genome-wide expression quantitative trait locus (eQTL) mapping. This is, however, only feasible with a reasonable sized population with gene expression data and with real or imputed SV genotypes. Some recent studies in humans have suggested that SV have larger effect sizes than SNPs and INDEL [69, 70]. In the last decade, multiple studies have predicted that SV have the potential to alter multiple adjacent genes: indeed, a recent estimation showed that SV-eQTL affect an average of 1.82 nearby genes, whereas SNP- and INDEL-eQTL only affect an average of 1.09 genes [46]. Thus, transcriptome changes induced by genomic SV are of strong interest to investigate. It should be noted that the molecular mechanism by which the Celtic and Friesian SV result in the polled cattle phenotype is still unknown, although a long RNA is suspected to be involved [71].

### Prediction of the theoretical impact of SV

There are many bioinformatic tools to predict the effect of SNPs, such as SIFT [72] and VEP [73]. Prediction of the effect of SV adds more complexity as there are different types of SV (such as insertions, deletions, and inversions) and they have the potential to influence the linear as well as the three-dimensional genome structure [74]. These different types of SV will need to be accounted for when predicting their effects. Several strategies to predict SV effects in humans have deployed existing tools to predict the biological effects of individual bases spanning the SV [75–77]. Theoretically, this strategy can also be applied to livestock species.

### Incorporation of SV discoveries to improve gene functional annotation

Multi-omics analyses including ATAC/ChIP/Iso-seq may be beneficial to explain the mechanism that underpins the effect of an SV (for an example, see [40]). Also, as described in previous sections, SV are of interest not only for the purpose of identifying simple mendelian mutations but also for their role in explaining variation in complex traits. At present, the FAANG (Functional Annotation of Animal Genomes) consortium is building livestock-specific genome-wide ‘OMICS’ resources to improve the functional annotation available for a range of species, tissues and developmental phases [78]. This type of annotation combined with the knowledge of SV could be used in prediction frameworks for the importance of a SV on complex traits similar to the FAETH score method used for SNPs and INDEL [79]. In addition, it is important to note that native methylation capturing is now available with both Oxford Nanopore and PacBio, so we believe that the analysis of multiple methylomes gathered from the large sequencing consortiums could provide a tremendous opportunity to further examine genomic imprinting or epigenetic marks [80]. Another question of interest is to examine if SV from specific genomic regions have very large effects on phenotypes. For example, SV within coding regions or regions enriched for sites that are conserved across vertebrates may result in large-effect SV associated with fitness. Interestingly, a recent study in bovine found evidence that SV were less likely to be located in “core” eukaryote genes [23] suggesting that there may be selective purging of SV in these genes due to highly detrimental effects. Of course, many SV will potentially encompass a range of genomic regions such as coding and non-coding. To assess the validity of predicted SV effects, one could compare the ranking of SV between predicted functional effects and SV genome-wide association studies (GWAS) results on complex traits such as fertility and survival.

## Application of the knowledge of SV location and genotype to improve genomic tools for animal breeding

Undoubtedly, post-validation and further downstream, there is still the ultimate question of how best to apply knowledge of the impact of SV to livestock breeding. For example: how common are functional SV, how accurately can they be imputed and/or incorporated for genotyping on a platform such as custom SNP panels, genotyping-by-sequencing or low-pass sequencing (using either short reads or long reads). Adopting SV in combination with SNV from both long/short read libraries to estimate the genomic heritability of quantitative traits is also of interest [23] and requires further investigation since long reads offer a higher resolution for SV, in addition to accurate phasing of long haplotypes and therefore better imputation. Last but not least, SV could be a target for the CRISPR gene editing technology that might provide benefits for specific situations to improve animal productivity, health or welfare outcomes (e.g., editing the poll trait in cattle [81] or other genetically improved livestock [82–84]). However CRISPR-like editing approaches require more active research to confirm their feasibility for application in livestock, because recent studies suggested that unintended off-target SV might be created as an artefact [85]

In the near future, it is within reach to build a collaborative multi-institutional long-read sequencing project (perhaps in conjunction with existing short-read consortiums) to build large-scale reference populations to enable the discovery and imputation of SV into large, genotyped populations of livestock. Either alone, or combined with imputed SNPs and INDEL, this would enable population-scale and GWAS with SV to determine the impact of SV on quantitative trait phenotypes as well as Mendelian traits. Furthermore, we can anticipate that the increasing availability of these resources in genomic prediction settings for a range of traits will deliver positive impacts for livestock breeding. In addition, most SNPs are commonly found to be biallelic (two observed alleles), while many SV can be multi-allelic (multiple observed alleles), as well as having slightly different breakpoints between individuals in large cohorts. Undoubtedly, these features create future challenges for analytical approaches [86]. Ideally, we would need thousands of animals in the reference population to accurately discover and impute SV for livestock breeding applications. In the initial phases it would likely be preferable to include parent–offspring trios to determine the accuracy of SV detection and phasing, as well as widely-used recent ancestors from a limited number of the most important breeds, while increasing the number of breeds in the future. The addition of more breeds will not only increase the diversity of the SV catalogue but would be useful to better understand the evolutionary and more recent history of SV, and in particular to understand if there has been some selective advantage for/against specific SV. It is also of interest to include suspected carrier/affected animals with deleterious conditions in an attempt to capture SV that may be responsible for these.

## Conclusions

Through this review, we provide a snapshot of the landscape of long-read sequencing in livestock and discuss the exciting developments for the discovery and application of SV. Significant ongoing technological improvements have paved the way to apply genome-wide long-read sequencing to population-scale projects. With this long-read technology, we can now dissect these structural variants with unprecedented detail as well as develop approaches to test their significance for key traits in livestock. We believe that although the generation and analyses of population-scale long-read sequencing data remains challenging in the next few years, now is the right time to start investing in multi-institutional collaborations that can integrate and use the huge volume of data generated from SNP array, short-read, and long-read technologies. We argue that a collaborative approach is a cost-effective proposal to more comprehensively and rapidly advance livestock genomics and that investment now will bring rewards in the near- to medium-term future.

## Data Availability

Not applicable.

## References

[1] Freeman JL, Perry GH, Feuk L, Redon R, McCarroll SA, Altshuler DM (2006). Copy number variation: new insights in genome diversity. Genome Res.

[2] Feuk L, Carson AR, Scherer SW (2006). Structural variation in the human genome. Nat Rev Genet.

[3] Ho SS, Urban AE, Mills RE (2020). Structural variation in the sequencing era. Nat Rev Genet.

[4] Liu GE, Ventura M, Cellamare A, Chen L, Cheng Z, Zhu B (2009). Analysis of recent segmental duplications in the bovine genome. BMC Genomics.

[5] Bickhart DM, Hou Y, Schroeder SG, Alkan C, Cardone MF, Matukumalli LK (2012). Copy number variation of individual cattle genomes using next-generation sequencing. Genome Res.

[6] Boussaha M, Esquerré D, Barbieri J, Djari A, Pinton A, Letaief R (2015). Genome-wide study of structural variants in bovine Holstein, Montbéliarde and Normande dairy breeds. PLoS One.

[7] Crysnanto D, Leonard AS, Fang Z-H, Pausch H (2021). Novel functional sequences uncovered through a bovine multiassembly graph. Proc Natl Acad Sci USA.

[8] Carvalho CMB, Lupski JR (2016). Mechanisms underlying structural variant formation in genomic disorders. Nat Rev Genet.

[9] Middelkamp S, Vlaar JM, Giltay J, Korzelius J, Besselink N, Boymans S (2019). Prioritization of genes driving congenital phenotypes of patients with de novo genomic structural variants. Genome Med.

[10] Beyter D, Ingimundardottir H, Oddsson A, Eggertsson HP, Bjornsson E, Jonsson H (2021). Long-read sequencing of 3,622 Icelanders provides insight into the role of structural variants in human diseases and other traits. Nat Genet.

[11] Sedlazeck FJ, Lee H, Darby CA, Schatz MC (2018). Piercing the dark matter: bioinformatics of long-range sequencing and mapping. Nat Rev Genet.

[12] Mahmoud M, Gobet N, Cruz-Dávalos DI, Mounier N, Dessimoz C, Sedlazeck FJ (2019). Structural variant calling: the long and the short of it. Genome Biol.

[13] De Coster W, Weissensteiner MH, Sedlazeck FJ (2021). Towards population-scale long-read sequencing. Nat Rev Genet.

[14] Amarasinghe SL, Su S, Dong X, Zappia L, Ritchie ME, Gouil Q (2020). Opportunities and challenges in long-read sequencing data analysis. Genome Biol.

[15] Karst SM, Ziels RM, Kirkegaard RH, Sørensen EA, McDonald D, Zhu Q (2021). High-accuracy long-read amplicon sequences using unique molecular identifiers with nanopore or PacBio sequencing. Nat Methods.

[16] Marx V (2021). Long road to long-read assembly. Nat Methods.

[17] Fadista J, Thomsen B, Holm L-E, Bendixen C (2010). Copy number variation in the bovine genome. BMC Genomics.

[18] Jenkins GM, Goddard ME, Black MA, Brauning R, Auvray B, Dodds KG (2016). Copy number variants in the sheep genome detected using multiple approaches. BMC Genomics.

[19] Bickhart DM, Xu L, Hutchison JL, Cole JB, Null DJ, Schroeder SG (2016). Diversity and population-genetic properties of copy number variations and multicopy genes in cattle. DNA Res.

[20] Yang L, Xu L, Zhou Y, Liu M, Wang L, Kijas JW (2018). Diversity of copy number variation in a worldwide population of sheep. Genomics.

[21] Henkel J, Saif R, Jagannathan V, Schmocker C, Zeindler F, Bangerter E (2019). Selection signatures in goats reveal copy number variants underlying breed-defining coat color phenotypes. PLoS Genet.

[22] Bickhart D, Liu G (2014). The challenges and importance of structural variation detection in livestock. Front Genet.

[23] Chen L, Pryce JE, Hayes BJ, Daetwyler HD (2021). Investigating the effect of imputed structural variants from whole-genome sequence on genome-wide association and genomic prediction in dairy cattle. Animals (Basel).

[24] Goodwin S, McPherson JD, McCombie WR (2016). Coming of age: ten years of next-generation sequencing technologies. Nat Rev Genet.

[25] Shafin K, Pesout T, Chang P-C, Nattestad M, Kolesnikov A, Goel S (2021). Haplotype-aware variant calling with PEPPER-Margin-DeepVariant enables high accuracy in nanopore long-reads. Nat Methods.

[26] Vergult S, Van Binsbergen E, Sante T, Nowak S, Vanakker O, Claes K (2014). Mate pair sequencing for the detection of chromosomal aberrations in patients with intellectual disability and congenital malformations. Eur J Hum Genet.

[27] Hampton OA, English AC, Wang M, Salerno WJ, Liu Y, Muzny DM (2017). SVachra: a tool to identify genomic structural variation in mate pair sequencing data containing inward and outward facing reads. BMC Genomics.

[28] Sethi R, Becker J, de Graaf J, Löwer M, Suchan M, Sahin U (2020). Integrative analysis of structural variations using short-reads and linked-reads yields highly specific and sensitive predictions. PLoS Comput Biol.

[29] Wang O, Chin R, Cheng X, Wu MKY, Mao Q, Tang J (2019). Efficient and unique cobarcoding of second-generation sequencing reads from long DNA molecules enabling cost-effective and accurate sequencing, haplotyping, and *de novo* assembly. Genome Res.

[30] Harewood L, Kishore K, Eldridge MD, Wingett S, Pearson D, Schoenfelder S (2017). Hi-C as a tool for precise detection and characterisation of chromosomal rearrangements and copy number variation in human tumours. Genome Biol.

[31] Chakraborty M, VanKuren NW, Zhao R, Zhang X, Kalsow S, Emerson JJ (2018). Hidden genetic variation shapes the structure of functional elements in *Drosophila*. Nat Genet.

[32] Chan S, Lam E, Saghbini M, Bocklandt S, Hastie A, Cao H (2018). Structural variation detection and analysis using Bionano optical mapping. Methods Mol Biol.

[33] Yuan Y, Chung CY, Chan TF (2020). Advances in optical mapping for genomic research. Comput Struct Biotechnol J.

[34] Wang Y, Zhao Y, Bollas A, Wang Y, Au KF (2021). Nanopore sequencing technology, bioinformatics and applications. Nat Biotechnol.

[35] Schatz M. Extended stats for maximum nanopore read lengths. https://github.com/schatzlab/long-read-commentary/blob/main/Nanopore_stats_extended.csv/. Accessed 21 Jan 2023.

[36] Rhoads A, Au KF (2015). PacBio sequencing and its applications. Genom Proteom Bioinform.

[37] Hon T, Mars K, Young G, Tsai Y-C, Karalius JW, Landolin JM (2020). Highly accurate long-read HiFi sequencing data for five complex genomes. Sci Data.

[38] Lal A, Brown M, Mohan R, Daw J, Drake J, Israeli J (2021). Improving long-read consensus sequencing accuracy with deep learning. BioRxiv.

[39] Murigneux V, Rai SK, Furtado A, Bruxner TJC, Tian W, Harliwong I (2020). Comparison of long-read methods for sequencing and assembly of a plant genome. GigaScience.

[40] Giani AM, Gallo GR, Gianfranceschi L, Formenti G (2020). Long walk to genomics: history and current approaches to genome sequencing and assembly. Comput Struct Biotechnol J.

[41] Wang T, Antonacci-Fulton L, Howe K, Lawson HA, Lucas JK, Phillippy AM (2022). The human pangenome project: a global resource to map genomic diversity. Nature.

[42] Ebler J, Ebert P, Clarke WE, Rausch T, Audano PA, Houwaart T (2022). Pangenome-based genome inference allows efficient and accurate genotyping across a wide spectrum of variant classes. Nat Genet.

[43] Hickey G, Heller D, Monlong J, Sibbesen JA, Sirén J, Eizenga J (2020). Genotyping structural variants in pangenome graphs using the vg toolkit. Genome Biol.

[44] Sirén J, Monlong J, Chang X, Novak AM, Eizenga JM, Markello C (2021). Pangenomics enables genotyping of known structural variants in 5202 diverse genomes. Science.

[45] Eizenga JM, Novak AM, Sibbesen JA, Heumos S, Ghaffaari A, Hickey G (2020). Pangenome graphs. Annu Rev Genom Hum Genet.

[46] Scott AJ, Chiang C, Hall IM (2021). Structural variants are a major source of gene expression differences in humans and often affect multiple nearby genes. Genome Res.

[47] Alonge M, Wang X, Benoit M, Soyk S, Pereira L, Zhang L (2020). Major impacts of widespread structural variation on gene expression and crop improvement in tomato. Cell.

[48] Mortazavi M, Ren Y, Saini S, Antaki D, St Pierre CL, Williams A (2022). SNPs, short tandem repeats, and structural variants are responsible for differential gene expression across C57BL/6 and C57BL/10 substrains. Cell Genom.

[49] Qanbari S (2019). On the extent of linkage disequilibrium in the genome of farm animals. Front Genet.

[50] Lee Y-L, Takeda H, Costa Monteiro Moreira G, Karim L, Mullaart E, Coppieters W (2021). A 12 kb multi-allelic copy number variation encompassing a GC gene enhancer is associated with mastitis resistance in dairy cattle. PLoS Genet.

[51] Kadri NK, Sahana G, Charlier C, Iso-Touru T, Guldbrandtsen B, Karim L (2014). A 660-Kb deletion with antagonistic effects on fertility and milk production segregates at high frequency in Nordic Red cattle: additional evidence for the common occurrence of balancing selection in livestock. PLoS Genet.

[52] Gabur I, Chawla HS, Snowdon RJ, Parkin IAP (2019). Connecting genome structural variation with complex traits in crop plants. Theor Appl Genet.

[53] Jeffares DC, Jolly C, Hoti M, Speed D, Shaw L, Rallis C (2017). Transient structural variations have strong effects on quantitative traits and reproductive isolation in fission yeast. Nat Commun.

[54] Chen L, Abel HJ, Das I, Larson DE, Ganel L, Kanchi KL (2021). Association of structural variation with cardiometabolic traits in Finns. Am J Hum Genet.

[55] Delahaye C, Nicolas J (2021). Sequencing DNA with nanopores: troubles and biases. PLoS One.

[56] Weirather J, de Cesare M, Wang Y, Piazza P, Sebastiano V, Wang X (2017). Comprehensive comparison of Pacific biosciences and Oxford nanopore technologies and their applications to transcriptome analysis. F1000Research.

[57] Zhang H, Jain C, Aluru S (2020). A comprehensive evaluation of long read error correction methods. BMC Genomics.

[58] Dohm JC, Peters P, Stralis-Pavese N, Himmelbauer H (2020). Benchmarking of long-read correction methods. NAR Genom Bioinform.

[59] Sereika M, Kirkegaard RH, Karst SM, Michaelsen TY, Sørensen EA, Wollenberg RD (2022). Oxford nanopore R10.4 long-read sequencing enables the generation of near-finished bacterial genomes from pure cultures and metagenomes without short-read or reference polishing. Nat Methods.

[60] Martin M, Patterson M, Garg S, Fischer SO, Pisanti N, Klau GW (2016). WhatsHap: fast and accurate read-based phasing. bioRxiv.

[61] Edge P, Bafna V, Bansal V (2017). HapCUT2: robust and accurate haplotype assembly for diverse sequencing technologies. Genome Res.

[62] Davies RW, Kucka M, Su D, Shi S, Flanagan M, Cunniff CM (2021). Rapid genotype imputation from sequence with reference panels. Nat Genet.

[63] Zhou Y, Leung AW-S, Ahmed SS, Lam T-W, Luo R (2022). Duet: SNP-assisted structural variant calling and phasing using Oxford nanopore sequencing. Bioinformatics.

[64] Lin J-H, Chen L-C, Yu S-C, Huang Y-T (2022). LongPhase: an ultra-fast chromosome-scale phasing algorithm for small and large variants. Bioinformatics.

[65] Pausch H, MacLeod IM, Fries R, Emmerling R, Bowman PJ, Daetwyler HD (2017). Evaluation of the accuracy of imputed sequence variant genotypes and their utility for causal variant detection in cattle. Genet Sel Evol.

[66] Low WY, Tearle R, Liu R, Koren S, Rhie A, Bickhart DM (2020). Haplotype-resolved genomes provide insights into structural variation and gene content in Angus and Brahman cattle. Nat Commun.

[67] Chaisson MJP, Sanders AD, Zhao X, Malhotra A, Porubsky D, Rausch T (2019). Multi-platform discovery of haplotype-resolved structural variation in human genomes. Nat Commun.

[68] van Arensbergen J, Pagie L, FitzPatrick VD, de Haas M, Baltissen MP, Comoglio F (2019). High-throughput identification of human SNPs affecting regulatory element activity. Nat Genet.

[69] Jakubosky D, D’Antonio M, Bonder MJ, Smail C, Donovan MKR, Young Greenwald WW (2020). Properties of structural variants and short tandem repeats associated with gene expression and complex traits. Nat Commun.

[70] Chiang C, Scott AJ, Davis JR, Tsang EK, Li X, Kim Y (2017). The impact of structural variation on human gene expression. Nat Genet.

[71] Allais-Bonnet A, Grohs C, Medugorac I, Krebs S, Djari A, Graf A (2013). Novel insights into the bovine polled phenotype and horn ontogenesis in Bovidae. PLoS One.

[72] Ng PC, Henikoff S (2003). SIFT: predicting amino acid changes that affect protein function. Nucleic Acids Res.

[73] McLaren W, Gil L, Hunt SE, Riat HS, Ritchie GRS, Thormann A (2016). The Ensembl variant effect predictor. Genome Biol.

[74] Shanta O, Noor A, Sebat J, Human Genome Structural Variation Consortium (HGSVC) (2020). The effects of common structural variants on 3D chromatin structure. BMC Genomics.

[75] Ganel L, Abel HJ, Hall IM, FinMetSeq Consortium (2017). SVScore: an impact prediction tool for structural variation. Bioinformatics.

[76] Kumar S, Harmanci A, Vytheeswaran J, Gerstein MB (2020). SVFX: a machine learning framework to quantify the pathogenicity of structural variants. Genome Biol.

[77] Danis D, Jacobsen JOB, Balachandran P, Zhu Q, Yilmaz F, Reese J (2022). SvAnna: efficient and accurate pathogenicity prediction of coding and regulatory structural variants in long-read genome sequencing. Genome Med.

[78] Andersson L, Archibald AL, Bottema CD, Brauning R, Burgess SC, Burt DW (2015). Coordinated international action to accelerate genome-to-phenome with FAANG, the functional annotation of animal genomes project. Genome Biol.

[79] Xiang R, van den Berg I, MacLeod IM, Hayes BJ, Prowse-Wilkins CP, Wang M (2019). Quantifying the contribution of sequence variants with regulatory and evolutionary significance to 34 bovine complex traits. Proc Natl Acad Sci USA.

[80] Ibeagha-Awemu EM, Zhao X (2015). Epigenetic marks: regulators of livestock phenotypes and conceivable sources of missing variation in livestock improvement programs. Front Genet.

[81] Young AE, Mansour TA, McNabb BR, Owen JR, Trott JF, Brown CT (2020). Genomic and phenotypic analyses of six offspring of a genome-edited hornless bull. Nat Biotechnol.

[82] Tait-Burkard C, Doeschl-Wilson A, McGrew MJ, Archibald AL, Sang HM, Houston RD (2018). Livestock 2.0—genome editing for fitter, healthier, and more productive farmed animals. Genome Biol.

[83] Kalds P, Zhou S, Cai B, Liu J, Wang Y, Petersen B (2019). Sheep and Goat genome engineering: From random transgenesis to the CRISPR era. Front Genet.

[84] Perisse IV, Fan Z, Singina GN, White KL, Polejaeva IA (2021). Improvements in gene editing technology boost its applications in livestock. Front Genet.

[85] Höijer I, Emmanouilidou A, Östlund R, van Schendel R, Bozorgpana S, Tijsterman M (2022). CRISPR-Cas9 induces large structural variants at on-target and off-target sites in vivo that segregate across generations. Nat Commun.

[86] Handsaker RE, Van Doren V, Berman JR, Genovese G, Kashin S, Boettger LM (2015). Large multiallelic copy number variations in humans. Nat Genet.

[87] Rothammer S, Capitan A, Mullaart E, Seichter D, Russ I, Medugorac I (2014). The 80-kb DNA duplication on BTA1 is the only remaining candidate mutation for the polled phenotype of Friesian origin. Genet Sel Evol.

[88] Menzi F, Besuchet-Schmutz N, Fragnière M, Hofstetter S, Jagannathan V, Mock T (2016). A transposable element insertion in APOB causes cholesterol deficiency in Holstein cattle. Anim Genet.

[89] Guo Y, Gu X, Sheng Z, Wang Y, Luo C, Liu R (2016). A complex structural variation on chromosome 27 leads to the ectopic expression of HOXB8 and the muffs and beard phenotype in chickens. PLoS Genet.

[90] Schütz E, Wehrhahn C, Wanjek M, Bortfeld R, Wemheuer WE, Beck J (2016). The Holstein Friesian lethal haplotype 5 (HH5) results from a complete deletion of TBF1M and cholesterol deficiency (CDH) from an ERV-(LTR) insertion into the coding region of APOB. PLoS One.

[91] Küttel L, Letko A, Häfliger IM, Signer-Hasler H, Joller S, Hirsbrunner G (2019). A complex structural variant at the KIT locus in cattle with the Pinzgauer spotting pattern. Anim Genet.

[92] Becker D, Weikard R, Schulze C, Wohlsein P, Kühn C (2020). A 50-kb deletion disrupting the RSPO2 gene is associated with tetradysmelia in Holstein Friesian cattle. Genet Sel Evol.

[93] Artesi M, Tamma N, Deckers M, Karim L, Coppieters W, Van den Broeke A (2020). Colour-sidedness in Gloucester cattle is associated with a complex structural variant impacting regulatory elements downstream of KIT. Anim Genet.

[94] Jacinto JGP, Häfliger IM, Letko A, Drögemüller C, Agerholm JS (2020). A large deletion in the COL2A1 gene expands the spectrum of pathogenic variants causing bulldog calf syndrome in cattle. Acta Vet Scand.

[95] Lamb HJ, Hayes BJ, Randhawa IAS, Nguyen LT, Ross EM (2021). Genomic prediction using low-coverage portable nanopore sequencing. PLoS One.

[96] Simon R, Lischer HEL, Pieńkowska-Schelling A, Keller I, Häfliger IM, Letko A (2020). New genomic features of the polled intersex syndrome variant in goats unraveled by long-read whole-genome sequencing. Anim Genet.

[97] O’Toole D, Häfliger IM, Leuthard F, Schumaker B, Steadman L, Murphy B (2021). X-Linked hypohidrotic ectodermal dysplasia in crossbred beef cattle due to a large deletion in EDA. Animals (Basel).

[98] Trigo BB, Utsunomiya ATH, Fortunato AAAD, Milanesi M, Torrecilha RBP, Lamb H (2021). Variants at the ASIP locus contribute to coat color darkening in Nellore cattle. Genet Sel Evol.

[99] Zhu T, Liu M, Peng S, Zhang X, Chen Y, Lv X (2022). A deletion upstream of SOX10 causes light yellow plumage colour in chicken. Genes (Basel).

[100] Zhao P, Li J, Kang H, Wang H, Fan Z, Yin Z (2016). Structural variant detection by large-scale sequencing reveals new evolutionary evidence on breed divergence between Chinese and European pigs. Sci Rep.

[101] Fève K, Foissac S, Pinton A, Mompart F, Esquerré D, Faraut T (2017). Identification of a t(3;4)(p1.3;q1.5) translocation breakpoint in pigs using somatic cell hybrid mapping and high-resolution mate-pair sequencing. PLoS One.

[102] Mesbah-Uddin M, Guldbrandtsen B, Iso-Touru T, Vilkki J, De Koning D-J, Boichard D (2017). Genome-wide mapping of large deletions and their population-genetic properties in dairy cattle. DNA Res.

[103] Couldrey C, Keehan M, Johnson T, Tiplady K, Winkelman A, Littlejohn MD (2017). Detection and assessment of copy number variation using PacBio long-read and Illumina sequencing in New Zealand dairy cattle. J Dairy Sci.

[104] Seol D, Ko BJ, Kim B, Chai H-H, Lim D, Kim H (2019). Identification of copy number variation in domestic chicken using whole-genome sequencing reveals evidence of selection in the genome. Animals (Basel).

[105] Mielczarek M, Frąszczak M, Giannico R, Minozzi G, Williams JL, Wojdak-Maksymiec K (2017). Analysis of copy number variations in Holstein–Friesian cow genomes based on whole-genome sequence data. J Dairy Sci.

[106] Kommadath A, Grant JR, Krivushin K, Butty AM, Baes CF, Carthy TR (2019). A large interactive visual database of copy number variants discovered in taurine cattle. GigaScience.

[107] Hu Y, Xia H, Li M, Xu C, Ye X, Su R (2020). Comparative analyses of copy number variations between *Bos taurus* and *Bos indicus*. BMC Genomics.

[108] Mei C, Junjvlieke Z, Raza SHA, Wang H, Cheng G, Zhao C (2020). Copy number variation detection in Chinese indigenous cattle by whole genome sequencing. Genomics.

[109] Butty AM, Chud TCS, Miglior F, Schenkel FS, Kommadath A, Krivushin K (2020). High confidence copy number variants identified in Holstein dairy cattle from whole genome sequence and genotype array data. Sci Rep.

[110] Upadhyay M, Derks MFL, Andersson G, Medugorac I, Groenen MAM, Crooijmans RPMA (2021). Introgression contributes to distribution of structural variations in cattle. Genomics.

[111] Du H, Zheng X, Zhao Q, Hu Z, Wang H, Zhou L (2021). Analysis of structural variants reveal novel selective regions in the genome of Meishan pigs by whole genome sequencing. Front Genet.

[112] Yuan C, Lu Z, Guo T, Yue Y, Wang X, Wang T (2021). A global analysis of CNVs in Chinese indigenous fine-wool sheep populations using whole-genome resequencing. BMC Genomics.

[113] Cumer T, Boyer F, Pompanon F (2021). Genome-wide detection of structural variations reveals new regions associated with domestication in small ruminants. Genome Biol Evol.

[114] Wang K, Hu H, Tian Y, Li J, Scheben A, Zhang C (2021). The chicken Pan-genome reveals gene content variation and a promoter region deletion in IGF2BP1 affecting body size. Mol Biol Evol.

[115] Leonard AS, Crysnanto D, Fang Z-H, Heaton MP, Vander Ley BL, Herrera C (2022). Structural variant-based pangenome construction has low sensitivity to variability of haplotype-resolved bovine assemblies. Nat Commun.

[116] Gao Y, Ma L, Liu GE (2022). Initial analysis of structural variation detections in cattle using Long-read sequencing methods. Genes (Basel).

[117] Li R, Gong M, Zhang X, Wang F, Liu Z, Zhang L (2022). The first sheep graph-based pan-genome reveals the spectrum of structural variations and their effects on tail phenotypes. bioRxiv.

[118] Zhou Y, Yang L, Han X, Han J, Hu Y, Li F (2022). Assembly of a pangenome for global cattle reveals missing sequences and novel structural variations, providing new insights into their diversity and evolutionary history. Genome Res.

[119] Bouwman AC, Derks MFL, Broekhuijse MLWJ, Harlizius B, Veerkamp RF (2020). Using short read sequencing to characterise balanced reciprocal translocations in pigs. BMC Genomics.

[120] Zhang J, Nie C, Li X, Zhao X, Jia Y, Han J (2022). Comprehensive analysis of structural variants in chickens using PacBio sequencing. Front Genet.

