# Correction: In it for the long run: perspectives on exploiting long-read sequencing in livestock for population scale studies of structural variants

**DOI:** 10.1186/s12711-023-00800-7

**Published:** 2023-04-11

**Authors:** Tuan V. Nguyen, Christy J. Vander Jagt, Jianghui Wang, Hans D. Daetwyler, Ruidong Xiang, Michael E. Goddard, Loan T. Nguyen, Elizabeth M. Ross, Ben J. Hayes, Amanda J. Chamberlain, Iona M. MacLeod

**Affiliations:** 1 Agriculture Victoria, AgriBio, Centre for AgriBioscience, Bundoora, VIC 3083 Australia; 2 School of Applied Systems Biology, La Trobe University, Bundoora, VIC 3083 Australia; 3 Faculty of Veterinary & Agricultural Science, The University of Melbourne, Parkville, VIC 3052 Australia; 4 Queensland Alliance for Agriculture and Food Innovation, University of Queensland, St Lucia, QLD 4072 Australia

**Correction: Genetic Selection Evolution (2023) 55:9** 10.1186/s12711-023-00783-5

After publication of original article [1], the authors identified two errors in the paper.

1. Under the heading ***Building long‑read reference populations for SV discovery, phasing and imputation***, point number 2, *“Existing short read databases with many sequenced individuals would continue to be invaluable for imputation of small sequence variants (e.g., 1000 Bull Genomes Project now includes over 9000 genomes)”*, there is an error in the number of genomes included in the current version 1000 Bull Genomes Project, i.e. the correct number is **6191** genomes in Run 9.
2. Reference 95 i.e. Lamb HJ, Hayes BJ, Randhawa IAS, Nguyen LT, Ross EM. Genomic prediction using low-coverage portable Nanopore sequencing. PLoS ONE. 2021;16:e0261274 cited in Table 1 for the polled allele in Brahman is incorrect. The correct reference 95 is supplied below:

Lamb HJ, Ross EM, Nguyen LT, Lyons RE, Moore SS, Hayes BJ. Characterization of the poll allele in Brahman cattle using long-read Oxford Nanopore sequencing. J Anim Sci. 2020;98:skaa127.
